# Understanding indirect requests for information in high-functioning autism

**DOI:** 10.1007/s10339-021-01056-z

**Published:** 2021-09-06

**Authors:** Eleonora Marocchini, Simona Di Paola, Greta Mazzaggio, Filippo Domaneschi

**Affiliations:** 1 Laboratory of Language and Cognition, Department of Humanities, University of Genoa, via Balbi 2, 2nd floor, Genoa, Italy; 2 Department of Educational Sciences, Psychology Unit, University of Genoa, Genoa, Italy; 3 Department of Humanities, University of Florence, Florence, Italy

**Keywords:** Experimental pragmatics, Indirect requests, High-functioning autism, Development, Theory of mind

## Abstract

Few works have addressed the processing of indirect requests in High-Functioning Autism (HFA), and results are conflicting. Some studies report HFA individuals’ difficulties in indirect requests comprehension; others suggest that it might be preserved in HFA. Furthermore, the role of Theory of Mind in understanding indirect requests is an open issue. The goal of this work is twofold: first, assessing whether comprehension of indirect requests for information is preserved in HFA; second, exploring whether mind-reading skills predict this ability. We tested a group of (*n* = 14; 9–12 years) HFA children and two groups of younger (*n* = 19; 5–6 years) and older (*n* = 28; 9–12 years) typically developing (TD) children in a semi-structured task involving direct, indirect and highly indirect requests for information. Results suggested that HFA can understand indirect and highly indirect requests, as well as TD children. Yet, while Theory of Mind skills seem to enhance older TD children understanding, this is not the case for HFA children. Therefore, interestingly, they could rely on different interpretative strategies.

## Introduction

The ability to perform and understand indirect speech acts is a core component of pragmatic competence (Searle 1979). A speech act is considered to be indirect when it is conveyed through the performance of another speech act. For instance, an utterance such as (1) directly performs the speech act of an assertion and indirectly conveys a request for information.

1. You have not sent me any updates on the project, lately.
  There might be various reasons for requests to be indirect: for the sake of politeness (Brown and Levinson 1987), for greater informativeness (Zufferey 2015) or to be deliberately ambiguous (Pinker 2007). For instance, a speaker who utters (2) can indirectly request for a specific information (an address) while explaining the reason for the request (the speaker does not remember it).
2. I forgot their address.
  Indirect requests (IRs) as the utterance in (2) are usually defined as non-conventionalized, since no conventional form (such as, for instance, a *Can you…?* form) is employed. These non-conventionalized IRs are also usually referred to as highly indirect requests (HIRs) or hints (Blum-Kulka 1987; Clark 1979). In fact, whether (2) counts as an IR depends on the particular context of utterance. For this reason, IRs are often conceptualized as an instance of non-literal language, along the lines of metaphor or irony, and their comprehension is often conceived and explained within the framework of conversational implicatures (see Green 2010; Terkourafi 2009), while some debate on whether conventionalized IRs such as *Can you…?* forms can be conceived as such is ongoing (Groefsema 1992; Ruytenbeek 2012—but see Ruytenbeek 2019; Yus 1999). Even though we did not test conventionalized IRs in the present study, the distinction between conventionalized and non-conventionalized IRs will be useful to explain some of the mixed results in the literature on IRs, and on IRs in Autism Spectrum Disorders (ASD) in particular.

Non-literal language comprehension has been widely studied in the ASD, providing mixed results up to now (more on this later). However, only a few studies were conducted on IRs. The present study will investigate IRs comprehension in ASD children, with a focus on IRs for information and the role of Theory of Mind (ToM) in their comprehension. We will first review the experimental literature on understanding IRs in typical development, then briefly outline the state of the art of pragmatics in ASD before reviewing experiments on IRs comprehension in ASD adults and children.

### IRs comprehension in typically developing children

The experimental literature on typical development (TD) seems to suggest that understanding forms of conventionalized IRs such as *Can you…?*, *May you…?*, *Would you…?*, and *Why don’t you…?* emerges early, from 2;6 years of age. For instance, Shatz (1978) tested 2-year-old children in a cooperative game in which the experimenter commented on some toy props and occasionally asked participants to perform some action with one of them. The experimenter’s requests could be presented in several forms that included IRs such as *Why don’t you…?* (e.g., *Why don’t you put the chair in the house?*). Children provided evidence that already at 2 years of age they seem to grasp the IR by behaving accordingly. Reeder (1980) tested 2; 6- and 3-years-olds’ IRs understanding in a paraphrases-choice task. Children were presented with a request such as *Would you like to do x?* and were asked to choose one out of 2 paraphrases that provided the interpretation of the request either as an indirect offer (e.g., *I’ll let you do x*), or as a request (e.g., *I want you to do x*). Data collected suggested that 3-year-olds performed better than 2; 6-year-olds in interpreting these forms of IRs.

Other studies exploring the developmental trajectory of IRs comprehension, testing different forms of IRs, provided a more precise view of the development of the phenomenon. Carrell (1981) asked 4–7-year-olds to color a circle either red or blue, following the experimenter’s requests. These could be formulated in 40 different ways, ranging from interrogatives such as *Can you color the circle blue?* to assertions such as *The circle really needs to be painted blue* and conditionals such as *I’ll be very happy if you make the circle blue*. Even though none of these might be properly defined as a hint, hence as a HIR, half of the IRs presented in this study displayed a positive surface form but conveyed a negative meaning—e.g., *Should you color the circle blue?* to suggest *Don’t color the circle blue. Color it red*. The results revealed that all age ranges were above chance in IRs understanding. However, a developmental pattern was observed: 4-year-olds provided 64.5% of correct interpretations, 5-year-olds 73.5%, 6-year-olds 78% and 7-year-olds 92%. Interestingly, with IRs that had a positive surface form and conveyed a negative meaning, 4- and 5-year-olds were at chance. The author links this pattern to children’s difficulty in understanding the mismatch between positive/negative surface forms and negative/positive conveyed meanings.

The findings on the developmental trajectory for IRs comprehension have been corroborated by other studies on highly indirect requests that used story completion tasks. Bernicot and Legros (1987) asked 3–4 and 5–6-years-olds to complete a story that ended with either a direct request or an IR. The interlocutor in the story would not comply with these. To complete the story, children could choose one out of 3 pictures, in which the speaker uttering the IR was shown very angry, unhappy, or okay. The type of the picture alternative selected by the children would provide an indication of the children’s ability to attribute to the speaker the intention of requesting. For instance, if the children chose the very angry characters, they were taken to attribute to the character a strong intention of request. The context was either strong (i.e., strongly biasing toward a directive interpretation) or weak (i.e., the object of the request was not explicitly mentioned or depicted). Five-to-six-year-olds performed better than the younger children and were sensitive to the context manipulation. However, they still exhibited greater difficulties in interpreting IRs as directives than direct requests. On the contrary, 3–4-year-olds seemed not to perceive any manipulation: they treated IRs similarly to direct requests and did not discriminate between contexts. According to the authors, younger children’s interpretation of IRs would not take into account the linguistic form of the request yet. Instead, children at this age would rely on the context, even though their ability to distinguish a strong vs. a weak context would not be mature enough yet.

Similar findings can be found in a study by Elrod (1987): children between 3; 3 and 6; 5 years of age were presented with illustrated stories that ended either with an IR (e.g., *Those cookies are for our guests tonight*) or a direct request (e.g., *Please don’t eat the cookies*). Children were then presented with three cards (and their verbal explanation) to complete the story with either a compliance to the IR (e.g., putting the cookie back); a non-compliance with a reference to the literal meaning of the IR (e.g., saying *These are for our guest* and eat a cookie); and a non-compliance without any reference to the literal meaning of the IR (e.g., eating a cookie). Elrod found that children younger than 5 years of age could not understand the IRs.

A similar developmental trajectory was found by Bucciarelli et al. (2003), who tested children between 2;6 and 7 years of age with a range of IRs (together with direct) that included conventionalized indirect (e.g., *Sorry, could you close the window?*) and highly indirect requests (e.g., *Excuse me, I am studying*, as a request to stop making noise). All age groups exhibited higher difficulties with HIRs than both direct and conventionalized IRs. Yet, 6–7-year-olds correctly interpreted HIRs 68% of the times, against the 43% of 4;6–5;6-year-olds, the 42% of 3;6–4-year-olds and the 38% of 2;6–3-year-olds. The authors associate children’s difficulty at interpreting HIRs with the complexity of the process of comprehension for such cases; the process, according to the authors, requires the ability to construct mental representations and build chains of inference, which increases with age. In addition to this, the development of IRs comprehension seems to unfold during school age, at least up to 8 years of age (Bernicot et al. 2007).

Overall, the literature on IRs comprehension in TD children seems to show relatively high construct validity, as most of the studies above operationalized their research questions with a cooperative task, which warrants children’s engagement with the task. However, some aspects are noteworthy that might constrain the external validity of these studies. For instance, some studies used purely metalinguistic tasks (e.g., a paraphrase-choice task in Reeder 1980) or presented the task by openly stating it would involve requests (e.g., Carrel 1981). Additionally, some of these studies, especially the earlier works, were conducted on a small sample size and this poses issues on the generalization of results.—see Table 5 in the appendix for experimental details of the studies. Nevertheless, this body of research suggests two main findings. First, children show some understanding of IRs already from around age 3. However, second, the development of the ability to interpret IRs, particularly highly indirect ones, seems to go well beyond pre-school years. In fact, it is only at around 7–8 years of age that children start mastering HIRs.

## Pragmatics in high-functioning autism

The Autism Spectrum Disorder (ASD) is a neurodevelopmental disorder characterized by a range of impairments that include social cognition and communication; its clinical picture can vary from deep to high-functioning autism (Zufferey 2015).

One of the central issues in the debate of pragmatic abilities in ASD is the understanding of what could explain the well-known impairments in some domain of pragmatics. Traditionally, this has been attributed to a deficit in Theory of Mind (ToM), that is also notably compromised in ASD, and which could impede inferring the others’ intentions and mental states (Baron-Cohen 2000; Baron-Cohen et al. 1985). In turn, this idea draws on a traditional view that considers pragmatics as a theory of the speaker’s meaning in context, i.e., an activity based on a process of intentions attribution and recognition, hence as a sub-module of ToM^1^ (Sperber and Wilson, 2002).

Overall, then, the role of ToM in pragmatic abilities is a largely debated issue in the pragmatic literature as well as in the experimental literature on ASD individuals’ pragmatic competence. To this purpose, more recently, some authors have also proposed a mitigated idea of pragmatics as a cognitive system that entirely relies on mind-reading abilities (Bosco et al. 2018; Domaneschi and Bambini 2020). Importantly, however, whether massively or not, the role of ToM in pragmatics—therefore, pragmatic competence in ASD—is central in the current debate. Yet, the extent to which this is so remains to be established.^2^

Experimental research on pragmatic competence in ASD has provided mixed results up to now. On the one hand, some studies suggest that ASD individuals exhibit problems with several pragmatic phenomena, such as figurative language and irony (Happé 1993), the recognition of Gricean maxims violation (Surian 1996), turn-taking (Curcio and Paccia 1987), the use of context for ambiguity resolution (Jolliffe and Baron-Cohen 1999) and humor (Ozonoff and Miller 1996). On the other hand, more recent studies provided evidence that some pragmatic abilities are preserved in ASD and HFA, such as resolving lexical ambiguity with contextual clues (Brock et al. 2008) and deriving scalar implicatures (Chevallier et al. 2010; Hochstein et al. 2017; Pijnacker et al. 2009; but also see Mazzaggio et al. 2021 for different results on scalar and ad hoc implicatures).^3^ Moreover, it was shown that vocabulary skills seem to predict metaphor comprehension better than ToM both in HFA and TD (Norbury 2005).

As for IRs, the existing literature is still scant. In what follows, we now provide an overview on the experiments that addressed IRs comprehension in HFA, in both adulthood and childhood.

### IRs comprehension in high-functioning autism

Similarly to other pragmatic impairments, research on IRs understanding in HFA provided mixed findings for both adults and children.

As for adults, some studies suggested that the ability to understand IRs is impaired in ASD. Paul and Cohen (1985) replicated Carrell (1981) and asked adult ASD participants (mental age: 4–7 years) and controls with an age-matched cognitive disability to color a circle either in blue or in red (e.g., *I’ll be happy if you color this circle blue*). The different forms of IRs (e.g., conventionalized and non-conventionalized) were made in two experimental sessions that could or could not explicitly inform participants that they would be presented with requests. In the structured session, participants were clearly instructed that the experimenter would tell them to color some circles. In the unstructured session, IRs were made throughout a conversation between the experimenter and the participants while they were drawing. ASD participants performed worse than controls in both sessions, but—contrary to the control group—they performed worse in the unstructured than in the structured session. Moreover, similarly to TD children in Carrell’s (1981) study, ASD participants exhibited particular difficulties with negative items such as *You shouldn’t color the house blue*: they tended to respond by choosing the color named rather than its opposite. Evidence for HFA individuals’ impairment with IRs was also found in Ozonoff and Miller (1996). In this study, HFA adults (vs. controls matched for age and IQ) saw short vignettes that ended with a question (e.g., *Can you see that house number?*). Items appeared in two contexts that could either elicit a literal interpretation of the question (i.e., a question on the interlocutor’s ability) or an interpretation of this as a conventionalized IR (e.g., tell me that house number). Participants could choose one response (out of 4). The indirect context was found easier than the literal context for both HFA individuals and the control group. However, HFA participants provided overall less correct responses than the control group in both contexts. Interestingly, in incorrect trials, independently of the context, HFA participants interpreted as indirect requests the questions that in fact were not an indirect request—which the authors explain as an impairment in using context and meaning appropriately.

Counterevidence is also available. Deliens et al. (2018) tested ASD adults and neurotypical controls in two act-out tasks on IRs and irony. In the IRs task, participants were presented with some colored shapes that appeared in a grid. Upon hearing some requests, they could either move the shapes around the grid or press a yes or no button. The requests were imperatives or conventionalized/non-conventionalized IRs in two interrogative forms (e.g., *Can you…* or *Is it possible to… move the blue circle to the left of the red triangle?*). ASD participants interpreted the conventionalized IRs as much as the control group. Instead, they interpreted non-conventionalized IRs directively more often than the control group. The authors concluded that IRs understanding seems to be preserved in ASD.

As for HFA children, the available results provide a more homogeneous picture. MacKay and Shaw (2004) tested HFA children and TD controls aged between 8 and 11 years in a task that assessed the understanding of both the “meaning” and the “intention” of IRs. Children heard short stories that could end with non-conventionalized IRs such as *That cake looks delicious.* Afterward, they were asked two questions, one about the surface meaning of the utterance (e.g., *What does x mean?*) and one about its intent (e.g., *Why A utters x rather than asking for x directly?*). Both HFA and TD children correctly understood the IRs. However, contrary to TD children, the HFA participants exhibited more difficulties at explaining the intent behind the IRs, for instance they used more *I don’t know*. The authors suggest that HFA children grasp an IR, but they cannot explain this either because of their linguistic competence or because of a pragmatic impairment. Within a naturalistic scenario that avoided metalinguistic tasks, Kissine et al. (2012) videotaped the behavior of ASD children aged 4;3–12;5 years while they were involved in interactions with adults, who uttered 4 types of requests: imperative (e.g., *Pour the milk!*), declarative (e.g., *You are going to put the bottle in your bag*; *You forgot the water in your bag*), interrogative (e.g., *Can you throw this in the bin?*) and sub-sentential (e.g., *Your place,* with the meaning of *Get back to your place*). It was found that ASD children complied well with all types of adults’ requests and this was taken as evidence in favor of the fact that IRs understanding seems preserved in HFA children. Yet, the authors acknowledge the possibility that HFA children’s interpretive strategies might be fairly simple, based on their participants’ very low IQ. Overall, the earlier literature on IRs comprehension in ASD seems to show some of the same weaknesses in validity as the literature on TD children. In fact, some studies used metalinguistic tasks, which might decrease construct validity (e.g., the question about intentions by Mackay and Shaw, 2004) or involved a small sample size (Paul and Cohen, 1985; Kissine et al. 2012), thus reducing the statistical power of the study—see Table 6 in the Appendix for the experimental details of these studies.

Using a more fine-grained experimental method, Kissine et al. (2015) provided very interesting results. A group of ASD children and control TDs were tested in a semi-structured task involving a toy called Mr. Potato Head. Participants were asked to “dress” Mr. Potato Head with different items, such as a hat, glasses, etc. In the first phase, the children were invited to put the hat on the toy with a highly indirect request (e.g., *Oh, he has no hat!*); in the following two phases, the same sentence was uttered as a comment on a picture by a collaborator and then by the experimenter, to make sure the utterance would not stimulate a bias to action regardless of the context. ASD children complied with the IR in the first phase and inhibited the directive interpretation in the other two, while TD children had difficulties in comprehension in the first phase. The authors conclude that IRs comprehension is preserved in ASD. However, we think that of such a conclusion might be argued. First, the two groups of participants were not age-matched (HFA: 7–12-year-olds; TD: 2;7–3;6-year-olds). As such, it is unclear whether HFA children’s better performance reflected age group-related differences rather than genuine IRs comprehension in ASD. Moreover, the authors explain the dissociation between the two age groups pointing to the possibility that the younger TD group might not have sufficient mind-reading abilities to comply with the semi-structured task. Crucially, no ToM measure was collected in this study. In our view, the authors’ remark is partly controversial. In fact, there are at least two alternatives: first, if it is true, as they apparently assume, that IRs comprehension relies on ToM, then we should expect this ability to be compromised in HFAs, as it is for their skills in other pragmatic phenomena that notably rely on ToM. However, this conflicts with Kissine et al.’s conclusion that IRs are preserved in HFA. Second, if IRs comprehension does not rely on ToM, then the observed group difference reflects a mere developmental effect. To put it simple, TD children performed worse than HFA children because they are younger; thus, not only their ToM skills but also their general cognitive functioning is not developed enough yet. Hence, there might be reasons to think that the authors’ conclusion that IRs are preserved in HFA might remain a speculation. To rule this issue out, developmental biases need to be avoided: TD and HFA children at the same developmental stage have to be tested. Moreover, a measure of their ToM ability should be collected in order to verify whether (un)preserved mind-reading skills can explain IRs understanding. In this respect, it is worth mentioning that only a few studies on IRs comprehension have taken ToM into account, which, however, did not involve ASD or TD children. In fact, these studies were conducted on healthy adults (Trott and Bergen 2018; van Ackeren et al. 2012) and on a range of clinical populations such as Alzheimer disease (Cuerva et al. 2001), traumatic brain injuries (Muller et al. 2010) and on right-hemisphere lesions (Champagne-Lavau and Joanette 2009). Crucially, they overall suggest that ToM might play a role in understanding IRs.

All experimental studies on IRs understanding referred to in this section are also schematically reported in the Appendix Tables 5, 6, 7 with details on population characteristics, experimental tasks and results.

## Research questions and predictions

The main goal of the present study is to investigate the role of mind-reading skills in IRs understanding in HFA. Kissine et al. (2015) seminal work opened to the interesting possibility that ToM might not explain IRs understanding and that such a pragmatic phenomenon is preserved in this atypical population. We want to extend the study of Kissine et al. (2015). In order to do so, we tested three groups of participants: a group of HFA children, a group of age-matched TDs and a younger group of TDs. We assessed the comprehension of indirect requests for information through a purposely designed task suitable for all groups of children. Previous findings on IRs in childhood suggest that IRs are harder than direct requests in terms of processing. The overall difficulty with the indirectness of a request is likely associated with a greater inferential work needed to derive the speaker’s illocutionary intention. For this reason, we manipulated the level of (in)directness of a request to make the inter-group differences emerge more clearly and we used three types of requests: direct, indirect and highly indirect. We expected to replicate previous findings on the direct vs. indirect distinction and to observe more clearly eventual inter-group differences on the highly indirect condition, involving higher inferential abilities. More precisely, we are interested in looking at possible differences between the HFA and the older TD groups, while we included a group of younger TD children to allow for a comparison with Kissine and colleagues’ results. In this respect, we expected differences between the two TD groups, reflecting a developmental pattern: the older TD group should exhibit less difficulties with indirect and highly indirect request than the younger TD group, reflecting their more developed general cognitive functioning. As for HFA, we reasoned as follows: if IRs understanding is compromised in HFA, then we should observe greater difficulties in the indirect and highly indirect condition as compared to the age-matched TD group. We do not make any specific speculation on the differences between the HFA group and the group of younger TDs because, for the aforementioned reasons, it is hard to establish if we would be in front of a general pattern of development or if different performances would genuinely hinge on IRs comprehension. In sum, the first research question is:

RQ1: Is the ability to comprehend IRs compromised in HFA? Are there any differences depending on the amount of inferential work required by the request?

As mentioned earlier, previous studies on other clinical populations observed a relationship between ToM abilities and IRs comprehension. Yet, importantly, no study to date has ever addressed the potential role of mind-reading skills in understanding IRs neither in TD nor in ASD children. As it is known, ToM abilities are compromised in HFA. Nevertheless, recent studies on HFA reveal that, while some pragmatic abilities are compromised as the result of impaired mind-reading skills (e.g., metaphor, irony, etc.), some others are preserved. Therefore, the second research question is:

RQ2: Does the impairment in ToM play a role in HFA individuals’ (un)preserved IRs comprehension?

We reasoned that, if IRs understanding hinges on ToM, then the comprehension of the indirectness of a request might feature in terms of a positive function of ToM abilities. The greater the inferential work to derive the speaker’s illocutionary intention, the greater the involvement of ToM. Therefore, based on this assumption, a clearer relationship between HFA individuals’ ToM abilities and their comprehension of highly indirect requests should emerge. In particular, since ToM is impaired in HFA, understanding highly indirect requests should be more problematic in this population.

## Methods

### Participants

Sixty-one Italian monolingual children between 5; 2 and 12 years of age participated in the experiment. Children were divided into three groups. The first group consisted of 14 children who received a diagnosis of high-functioning autism (age range: 9–12 years; mean age(SD) = 10.6 (1.17); 2 F). They had all received their diagnosis of HFA (according to the DSM-IV criteria) by a team of trained neuropsychologists. The second group was composed of 28 typically developing children (TD), who were age-matched with the HFA children (age range: 9–12 years; mean age (SD) = 11.03(0.61); 11 F). The third group consisted of 19 younger TD children (age range: 5; 2–6; 3 years; mean age (SD) = 5.35(0.48); 6 F). All children were also tested for structural language through a standardized test for Italian, the *Batteria per la Valutazione del Linguaggio* (BVL_4-12) test for children aged 4–12 years (Marini et al. 2015). Participants in the HFA group were recruited in an ASD support center located in Genoa. Participants in the TD group were recruited in a primary school in Vicenza. The younger TD children were recruited in a kindergarten in Genoa. All participants were tested in a quiet area of their support center/school. None of them had neurological, linguistic, or hearing disorders.

Written informed consent was obtained from the parents/guardians of the participating children; since the Ethics Committee of our institution had not been constituted yet, at the time of the experiment, we could not ask for their approval. Nonetheless, the research has been conducted in full compliance with the ethical standards of APA ethical guidelines as well as of the 1964 Declaration of Helsinki.

### Materials and procedure

Children were administered four tasks: an IRs comprehension task; one task that assessed for their linguistic abilities; and two ToM tasks. The order of administration of the tasks was randomized across children to control for biases related to the order of the tasks. For each child, the overall experimental session was about 30 min long.

#### The IRs comprehension task

To test IRs understanding, we designed a semi-structured task in which children were presented with a drawing and were asked to help the experimenter to recreate it. A familiarization trial was first administered to accustom participants with the procedure and the task. In this phase, the experimenter showed to the child a drawing portraying some people and animals in a garden. She explained to the child that she would create a copy of the drawing. In order to do so, the child was instructed that the experimenter would not look at the original drawing and that the child would do this to help the experimenter. Throughout the familiarization phase, the experimenter uttered general questions, such as *Are there any children in the drawing?*

In the experimental phase, the child was shown the drawing of a farm and, again, was asked to help the experimenter to recreate this (e.g., *Now, I’ll try and redraw it [the drawing of the farm]. But I need your help because I can’t look at it while I draw*). The drawing portrayed a farm composed of a hen-house, a barn, a pond, a fence, an apple tree, and a tractor, along with several animals (a horse, a donkey, a cow, a cat, a dog, several sheep, pigs, chicken, chicks, and ducks). Throughout the session, the experimenter requested to the child for information on the elements portrayed in the drawing.

In total, 36 requests for information were created. These were presented in three conditions (12 per condition): Direct (DIR), such as *What color is the grass?*; Indirect (IR), such as *I don’t remember the color of the grass*; and Highly Indirect (HIR), such as *The color of the grass is hard to remember* (for all experimental items see the Supplementary Material hosted on the Open Science Framework web platform: https://osf.io/p2dn5). This manipulation generated three levels of indirectness of the request—Direct, Indirect and Highly Indirect—that, respectively, involved increasing inferential efforts to recognize the speaker’s illocutionary intention. We reasoned that to correctly interpret an indirect request such as *I don’t remember the color of the grass,* children needed to undergo an inferential path along the following lines: (i) *the experimenter does not remember the color, therefore* (ii) *she is probably requesting me to tell her what color it is*. Similarly, to comply with a highly indirect request such as *The color of the grass is hard to remember,* children needed to go through an inferential path that involved at least one more step: (i) *the experimenter states that the color is hard to remember*, (ii) *because she does not remember it herself,* and (iii) *since she does not remember this, she is probably requesting me to tell her*. Materials were divided into three lists such that the same child never saw the same item in all three conditions. Each child saw 12 requests/trials (4 per condition).

A set of filler items (N. 12) was also included. This consisted of yes/no questions of different syntactic complexity (e.g., *Is there a child in the farm?*; *Is the cat on the right of the drawing close to the donkey?*). The experimenter would also occasionally make remarks (e.g., *I will let you look at it when it’s done*) to ensure that children would have not be biased to react to every input from the experimenter. The order of administration of all items, experimental and filler, was randomized across participants.

Both the choice to present children with requests for information rather than requests for action and to add fillers and occasional remarks (which would not require a response) were made in the attempt to find a fair balance between the external validity of the experiment and the construct validity of the task. The aim was to avoid children’s known bias-to-action (Shatz, 1978) while maintaining as much as possible the ecology and engagement level of a typical act-out task. In fact, we designed our task in order to test more items per condition and different degrees of indirectness, contrary to Kissine et al. (2015) and most of the previous literature on the phenomenon. However, this would have created a potential bias-to-action if the response required from children was an action. Therefore, we designed our task so that children were required to respond with speech rather than with an action. In fact, in our task children did not have to touch or move the colored pencils. This avoided that their reactions to requests could be ascribed to a bias-to-action or fascination/familiarity to the colors in question. Importantly, the task and the experimental setting were implemented within a cooperative context between the child and the experimenter, in which the remarks served the purpose of keeping the child engaged and make the interaction as much natural as possible.

For the same reason, we recorded children during the task and scored children’s accuracy in the target items by listening to it at a later stage. Correct answers were given accuracy 1, and incorrect responses were given accuracy 0, following a common procedure in experimental research in pragmatics (e.g., Di Paola et al. 2020). A child’s response was considered as correct if the child correctly named the color that the experimenter requested for. In all other cases (e.g., the child did not provide any answer or provided an irrelevant answer), the child’s response was deemed incorrect.

#### Assessment of linguistic abilities

The *Batteria per la Valutazione del Linguaggio* (BVL) test (Marini et al. 2015) was used to assess participants’ linguistic competence. This is a standardized test for Italian that assesses children’s morphosyntactic abilities. In each of 40 trials, upon hearing a sentence uttered by the experimenter, the child is shown 4 pictures and is asked to point to the picture that best matches the experimenter’s utterance. The sentences vary in syntactic complexity (e.g., from *The boy reads the book* to *The mouse that the cat is chasing has cheese in its mouth*). Following the standardized scoring procedure for the test, each correct answer was assigned one score. A child’s response was deemed correct if the child pointed to the picture referred to by the experimenter’s utterance. The test score is obtained by counting the number of correct trials. The maximum possible score was 40, and the minimum possible score was 0.

#### Assessment of ToM abilities

Children’s ToM abilities were assessed using an Italian adaptation (Panzeri et al. 2020) of *Task F* and *Task I* of the Theory of Mind Task Battery (Hutchins et al. 2014). These are two false-belief tasks that tap first- (Task F) and second-order ToM (Task I). In both tasks, the experimenter told the child an illustrated story, at the end of which the child was asked three questions: a memory question, a false-belief question, and a control one asking for an explanation of the answers to the previous questions.

The first-order ToM task is a story with two characters, Antonio and Sonia. Antonio puts a book on a table and leaves. While he is away, Sonia comes in and puts the book into a drawer. At this point, Antonio comes back. The false-belief question asks for the location in which Antonio would look for the book.

The second-order ToM task consists of a story with three characters: Enrico, his mother and his grandfather. The mother tells Enrico he is not going to get the bike he wanted for his birthday, but a new pair of rollers. Later in the story, Enrico sees the bike hidden in a wardrobe and he says to himself that his mum did not in fact get him the rollers, but the bike. The mum did not see that Enrico found the bike. At this point of the story, the participant is asked what Enrico thinks he will get for his birthday, and whether his mother is aware that he knows he will get the bike. If the participant answers correctly, the last part of the story is presented. Here, the grandfather comes in and asks Enrico’s mother what Enrico thinks he will get for his birthday. In the end, the false-belief question is presented and the participant is asked what Enrico’s mother would reply to the grandfather (false-belief question).

In both tasks, if the child failed to answer the questions at first, the experimenter elicited these by showing four picture choices (for the first-order task: the picture of the table, the drawer, and two fillers, i.e., a chair and a desk with small drawers; for the second-order task: the rollers, the bike, and two fillers, i.e., a basket-ball and a baseball glove). The first-order false-belief task was administered first. If children did not pass it, they were not administered the second-order ToM task. We followed the standardized scoring procedure for this task. Children could either pass or fail the two false-belief tasks. For each of the tasks, if they passed this, they were assigned one point. If they failed this, they were assigned no point. In each of the two tasks, a child was assigned a pass if she correctly responded to all questions (i.e., the memory question, the false-belief question, and the control question).

### Coding and statistical analyses

All experimental sessions were audio-recorded, and children’s responses in the three tasks were subsequently coded by the experimenter.

Three types of statistical analyses were conducted. First, children’s accuracy in the IRs tasks was analyzed. Second, group-related differences in the BVL and the two ToM tasks were analyzed. Third, an analysis of predictors was conducted to assess whether children’s linguistic and/or mind-reading abilities play a role in their understanding of IRs.

Children’s accuracy in the IRs task was analyzed with Linear-Mixed Models statistics (LMMs), using the lme4 (Bates et al. 2015) and the lmerTest packages in the R environment, to provide F statistics with degrees of freedom. Tukey contrasts with the R package emmeans were used for post hoc comparisons. For each child, a composite score in each of the three experimental conditions of the IRs task was computed by counting the number of correct trials.^4^ In each condition, each child could be assigned a composite score between 0 and 4, i.e., each child had three composite scores between 0 and 4 (one for the direct, one for the indirect, and one for the highly indirect requests). The fixed-effects structure of the LMM model included Group (HFA, TD, and younger TD) and Condition (DIR, IR, HIR), along with the resulting interactions. The random structure of the model included random intercepts for subjects.

Group differences in structural language and mind-reading skills were analyzed with the Kruskal–Wallis rank sum test statistics, and Dwass–Steel–Critchlow–Fligner contrasts for pairwise comparisons.

As for the analysis of predictors, a LMM statistics was carried out in which participants’ composite score in the IRs task was the outcome variable and participants’ scores in the structural language task (the BVL) and in the ToM tasks were treated as predictors, together with Group, Condition and the resulting interactions. For each child, a composite ToM score was computed by counting the score in each of the two false-belief tasks.^5^ This returned 3 possible outcome scores: 0, if the child failed both tasks; 1, if the child passed only one of the two tasks; and 2, if the child passed both tasks.

All statistical analyses were conducted using R software (R Core Team, 2020). Data and code used for the analyses are available in the Supplementary Material hosted on the Open Science Framework web platform (link to the project: https://osf.io/p2dn5).

## Results

**Fig. 1:**
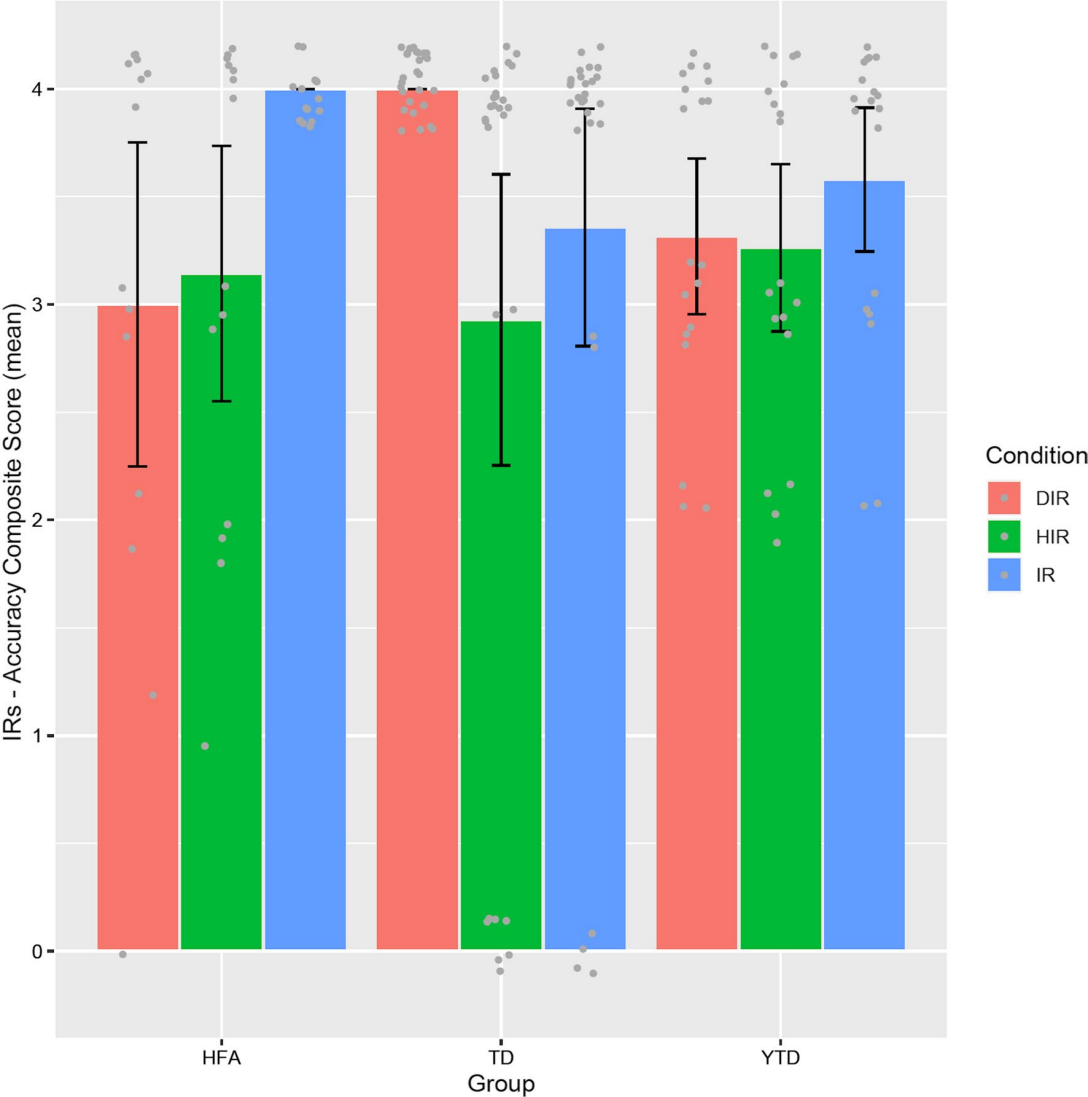
Mean composite score for the accuracy in the IRs task in each experimental group and condition. Error bars indicate the standard deviation. Jittered points indicate the individual data points. (HFA: High-Functioning Autism group; TD: older Typically Developing group; YTD: Younger Typically Developing group)

Table 1 reports children’s composite scores in the IRs comprehension task, together with children’s scores in the BVL test and in the assessments for ToM (i.e., first- and second-order ToM tasks, and composite ToM score). In addition, for a clearer illustration, Fig. 1 graphically displays the pattern of results in IRs comprehension across groups and conditions.

**Table 1:** Mean score (SD) of each experimental group in the tasks assessing for IRs comprehension (conditions DIR, IR and HIR), linguistic (BVL) and mind-reading abilities (first- and second-order ToM; and ToM composite score)

	TD children	HFA children	Younger TD children
*IRs composite score:*			
DIR	4.00 (0)	3.00 (1.30)	3.32 (0.74)
IR	3.36 (1.41)	4.00 (0)	3.58 (0.69)
HIR	2.93 (1.74)	3.14 (1.02)	3.26 (0.80)
BVL score	37.3 (2.32)	30.1 (6.14)	29.2 (3.75)
First-order ToM	0.92 (0.26)	0.50 (0.51)	0.57 (0.50)
Second-order ToM	0.71 (0.46)	0.35 (0.49)	0.05 (0.22)
ToM composite score	1.64 (0.62)	0.85 (0.66)	0.63 (0.59)

### IRs comprehension

Overall, all children performed well in the IRs task (see Fig. 1). Yet, their performance does not seem the same independently of group and condition. The LMMs statistics (see Table 2 for all statistical details) revealed a significant main effect of condition, thus indicating that children provided significantly more correct responses to indirect than highly indirect requests (mean composite score (SD) in each condition: DIR: 3.55 (0.84); IR: 3.57 (1.05); HIR: 3.08 (1.34)). Additionally, and most important, the interaction Group by Condition was significant, too, and simple effects analysis (Table 2) revealed interesting group differences. First, older TD children exhibited significantly higher accuracy in direct than both indirect and highly indirect requests. Second, an opposite pattern emerged for HFA children, who provided significantly more correct responses to indirect than both highly indirect and direct requests, while no difference emerged between DIR and HIR in this group. Finally, no significant differences of condition emerged in the group of younger TD children.

**Table 2:** Results—Children’s accuracy in the IRs task

*A. F statistics with degrees of freedom*						
Condition		F (2.116) = 4.47; *p* = 0.01*				
Group		F (2.58) = 0.02; *p* = 0.97				
Condition X Group		F (4.116) = 4.39; *p* = 0.002**				
*Fixed-effects parameters of the Linear Mixed-Model*						
Coefficient		Estimate (*B*)	Std. error (*B*)	DF	*t*	*p*
Intercept		3.000	0.288	159.122	10.407	< 0.0001***
Condition HIR		0.142	0.360	116	0.396	0.692
Condition IR		1.000	0.360	116	2.771	0.006**
Group TD		1.000	0.353	159.120	2.832	0.005**
Group Younger TD		0.315	0.379	159.120	0.831	0.407
Cond HIR: Group TD		− 1.214	0.442	116	− 2.747	0.006**
Cond IR: Group TD		− 1.642	0.442	116	− 3.717	0.0003***
Cond HIR: Group Younger TD		− 0.195	0.475	116	− 0.411	0.681
Cond IR: Group Younger TD		− 0.736	0.475	116	− 1.549	0.124
*Likelihood-ratio Test: null model versus full model*						
Model		AIC	logLik	*χ*^2^	DF	*p*_(*χ*2)_
Null model		562.46	− 278.23			
Full model		551.99	− 265.00	26.468	8	0.0008***

*B. Pairwise comparisons for the main effect of condition*						
Comparison		B	SE	DF	*t*	*p*
DIR versus IR		− 0.207	0.18	116	− 1.149	0.48
DIR versus HIR		0.327	0.18	116	1.818	0.16
IR versus HIR		− 0.534	0.18	116	− 2.967	0.01*

*C. Simple effects analysis of condition across groups*						
Group	Comparison	B	SE	DF	*t*	*p*
TD	DIR versus IR	0.642	0.255	116	2.519	0.03*
	DIR versus HIR	1.071	0.255	116	4.198	0.0002***
	IR versus HIR	− 0.42 8	0.255	116	− 1.679	0.21
Younger TD	DIR versus IR	− 0.26	0.31	116	− 0.84	0.67
	DIR versus HIR	0.05	0.31	116	0.17	0.98
	IR versus HIR	− 0.31	0.31	116	− 1.01	0.56
HFA	DIR versus IR	− 1.00	0.361	116	− 2.771	0.01*
	DIR versus HIR	− 0.142	0.361	116	− 0.396	0.91
	IR versus HIR	− 0.857	0.361	116	− 2.375	0.04*

A. F statistics with degrees of freedom and details of the fixed-effects parameters from the Linear Mixed-Model; Likelihood-ratio test to assess the goodness of fit of the LMM model: comparison between a full model (i.e., all predictors in the fixed-effects structure and all random parameters in the random effects structure) and a null model (i.e., containing only the random parameters in the random effects structure)

B. Pairwise comparisons for the main effect of condition (Tukey contrasts)

C. Simple effects analysis of condition across groups (i.e., Tukey contrasts) to break down the significant GroupXCondition interaction

*p ≤ 0.05

**p ≤ 0.01

***p ≤ 0.001

Taken together, these data suggest different patterns of IRs comprehension in the three groups of participants.

### Group differences in BVL and ToM tasks

Significant differences emerged in children’s score in the BVL test depending on group: children in the older TD group scored significantly higher than both HFA children and children in the younger TD group. No differences emerged between HFA and younger TD children—see Table 3 for all statistical details.

**Table 3:** Results: Group differences in BVL and ToM Tasks. Kruskal–Wallis rank sum test for the effect of group in BVL score and children’s scores in all ToM measures (i.e., first-order ToM; second-order ToM; composite ToM score). Dwass–Steel–Critchlow–Fligner contrasts for pairwise comparisons between groups in the measures for BVL and ToM

Measure	Kruskal–Wallis rank sum test	Dwass–Steel–Critchlow–Fligner contrasts	
*BVL: Group differences*			
	*χ*^2^ (2) = 35.5; *p* < 0.001***; *ε*^2^ = 0.592	HFA versus TD	*W* = 5.91; *p* < 0.001***
		HFA versus Younger TD	*W* = − 1.24; *p* = 0.65
		TD versus Younger TD	*W* = − 7.73; *p* < 0.001***
*ToM: Group differences*			
1st order	*χ*^2^ (2) = 11.1; *p* = 0.004**; *ε*^2^ = 0.185	HFA versus TD	*W* = 4.45; *p* = 0.005**
		HFA versus Younger TD	*W* = 0.62; *p* = 0.89
		TD versus Younger TD	*W* = − 4.02; *p* = 0.005**
2nd order	*χ*^2^ (2) = 20.3; *p* < 0.001***; *ε*^2^ = 0.1338	HFA versus TD	*W* = 3.11; *p* = 0.072
		HFA versus Younger TD	*W* = − 3.12; *p* = 0.07
		TD versus Younger TD	*W* = − 6.26; *p *< 0.001***
Composite score	*χ*^2^ (2) = 23.1; *p* < 0.001***; *ε*^2^ = 0.385	HFA versus TD	*W* = 4.84; *p* = 0.002**
		HFA versus Younger TD	*W* = − 1.39; *p* = 0.587
		TD versus Younger TD	*W* = − 6.27; *p* < 0.001***

As for ToM, significant group differences emerged for the first-order ToM task: as with the BVL test, children in the older TD group scored significantly higher than both the HFA group and the younger TD group, while the HFA group and the younger TD group did not significantly differ (see Table 3 for the statistical details). Instead, the pattern of results

slightly changed for the second-order ToM task (Table 3). Significant group differences emerged, with older TD children scoring significantly higher than younger TD children. Yet, this time, the difference between older TD children and HFA children only approached significance, and so did the difference between HFA and younger TD children, too.

Most important, when children’s scores in the two ToM tasks were combined into a composite score to obtain an overall measure for ToM, the significant main effect of group was confirmed and TD children exhibited overall better ToM skills than both HFA and the younger TD children. On the contrary, this time, no significant difference was found between HFA children’s scores and those of the younger TD children—Table 3.

### Analysis of predictors

**Fig. 2:**
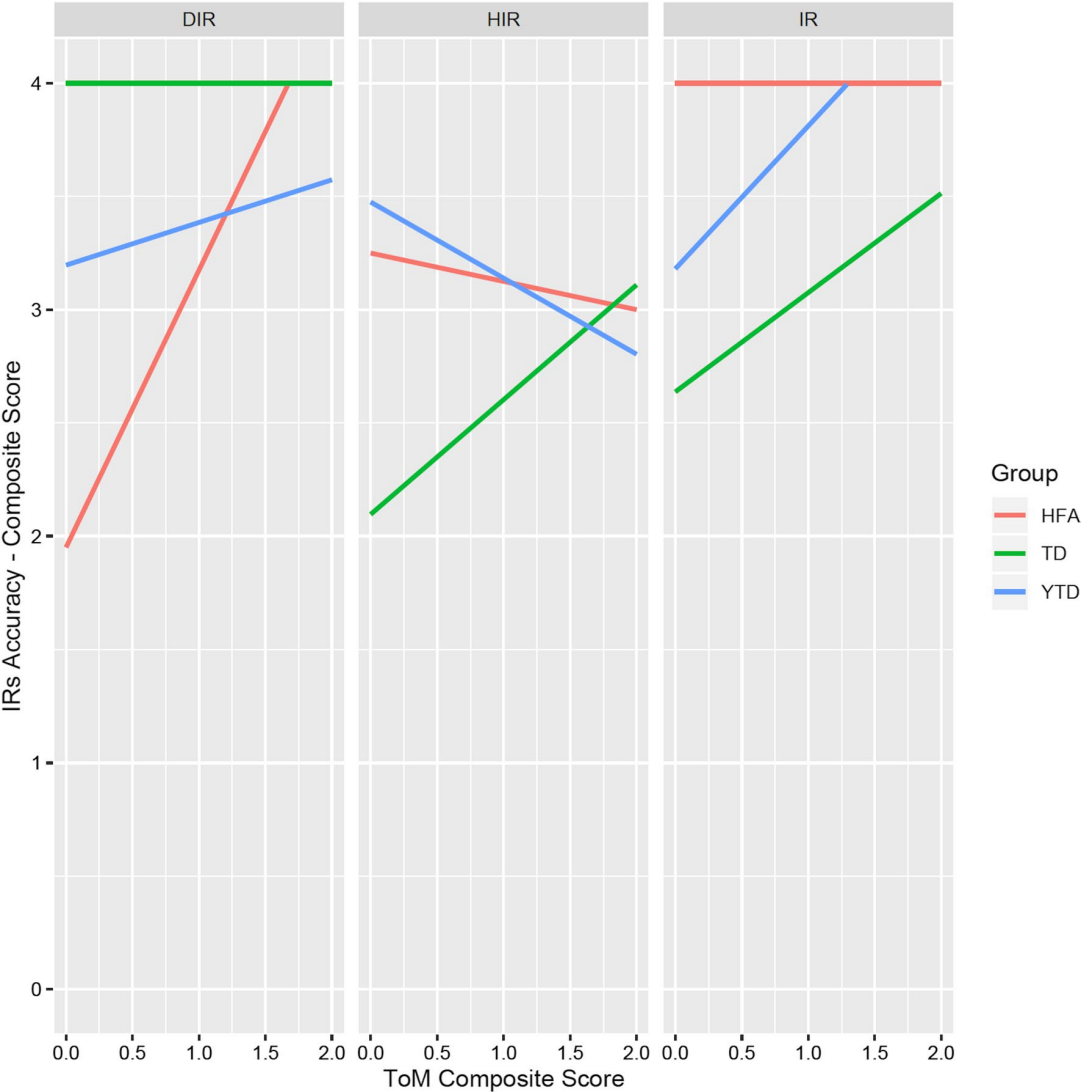
Mean composite score for the accuracy in the IRs task and participants’ composite scores in ToM, across groups and conditions (HFA: High-Functioning Autism group; TD: older Typically Developing group; YTD: Younger Typically Developing group)

All results from the LMMs statistics for the analysis of predictors are reported in Table 4. This analysis confirmed that children’s scores in the IRs comprehension task are significantly predicted by the type of request: when this is an IR, but not a HIR (vs. DIR), the effect is significant (i.e., significant effect of Condition IR only). Most interesting, children’s linguistic abilities as assessed by the BVL test did not reveal a significant predictor of IRs comprehension (i.e., no significant BVL-related effects). Even more interesting, mind-reading skills differently predicted the scores in the IRs comprehension task depending on participants’ group and the type of request. In fact, even though ToM marginally predicted children’s accuracy in the IRs task regardless of group, this revealed a significant predictor of children’s performance in the IRs task only in the group of older TDs and only with HIRs (i.e., significant 3-ways interaction Condition HIRX Group TD X ToM Composite score)—see Table 4 and Fig. 2.

**Table 4:** Results: Analysis of predictors. Fixed-effects parameters from the Linear Mixed-Model and Likelihood-ratio test to assess the goodness of fit of the LMM model: comparison between a full model (i.e., all predictors in the fixed-effects structure and all random parameters in the random effects structure) and a null model (i.e., containing only the random parameters in the random effects structure)

Coefficient	Estimate (*B*)	Std. error (*B*)	DF	*t*	*p*
*Fixed-effects parameters of the Linear Mixed-Model*					
Intercept	− 0.309	1.518	140.800	− 0.204	0.838
Condition HIR	2.707	1.881	104	1.439	0.153
Condition IR	4.309	1.881	104	2.291	0.024*
Group TD	4.309	3.822	140.800	1.128	0.261
Group Younger TD	4.259	2.724	140.800	1.563	0.120
BVL	0.084	0.054	140.800	1.569	0.118
ToM Composite	0.879	0.500	140.800	1.758	0.080
Cond HIR: Group TD	− 4.555	4.735	104	− 0.962	0.338
Cond IR: Group TD	− 1.159	4.735	104	− 0.245	0.807
Cond HIR: Group Younger TD	− 2.191	3.376	104	− 0.649	0.517
Cond IR: Group Younger TD	− 4.415	3.376	104	− 1.308	0.193
Group TD: BVL	− 0.084	0.105	140.800	− 0.805	0.422
Group Younger TD: BVL	− 0.112	0.099	140.800	− 1.139	0.256
Cond HIR: BVL	− 0.052	0.066	104	− 0.789	0.432
Cond IR: BVL	− 0.084	0.066	104	− 1.266	0.208
Group TD: ToM Composite	− 0.879	0.603	140.800	− 1.458	0.147
Group Younger TD: ToM Composite	− 0.588	0.722	140.800	− 0.815	0.416
Cond HIR: ToM Composite	− 1.134	0.619	104.000	− 1.831	0.070
Cond IR: ToM Composite	− 0.879	0.619	104.000	− 1.419	0.159
Cond HIR: Group TD: BVL	0.051	0.130	104.000	0.394	0.694
Cond IR: Group TD: BVL	− 0.033	0.130	104.000	− 0.254	0.800
Cond HIR: Group Younger TD: BVL	0.043	0.122	104.000	0.358	0.720
Cond IR: Group Younger TD: BVL	0.088	0.122	104.000	0.718	0.474
Cond HIR: Group TD: ToM Composite	1.640	0.747	104.000	2.195	0.030*
Cond IR: Group TD: ToM Composite	1.244	0.747	104.000	1.664	0.099
Cond HIR: Group Younger TD: ToM Composite	0.642	0.895	104.000	0.718	0.474
Cond IR: Group Younger TD: ToM Composite	1.309	0.895	104.000	1.463	0.146
*Likelihood-ratio test: null model versus full model*					
Model	AIC	logLik	*χ*^2^	DF	*p*_(* χ*2)_
Null model	562.46	− 278.23			
Full model	565.22	− 253.61	49.233	26	0.003**

Overall, then, children’s performance in the ToM tasks seems to play a role in IRs comprehension only for TD children (as compared to HFA children and the younger TD children) and when highly indirect requests are involved.

## Discussion

This study addressed the two following research questions:

RQ1: Is the ability to comprehend IRs compromised in HFA? Are there any differences depending on the amount of inferential work required by the request type?

RQ2: Does the impairment in ToM play a role in HFA individuals’ (un)preserved IRs comprehension?

In order to address RQ1, we assessed IRs comprehension in a group of HFA children, a group of age-matched TD controls and a group of younger TD children. In order to address RQ2, we assessed participants’ linguistic and mind-reading abilities. Two main patterns of results emerged. First, all groups exhibited a good understanding of all the request types. Yet, different comprehension patterns emerged depending on group: older TD children were fairly accurate with all types of requests, but still their accuracy was lower for both indirect and highly indirect requests (vs. direct requests); HFA children exhibited a different pattern: even though correctly interpreting also direct and highly indirect requests, they were more accurate with indirect requests; as for younger TD children, instead, no differences emerged based on the type of requests. Second, ToM predicted the older TD children’s ability to comprehend highly indirect requests: children who scored higher in ToM also were more accurate in HIRs comprehension. We now discuss separately these patterns of results, in relation to the RQs 1 and 2.

Starting from the first finding, overall children performed well in an act-out task that required interpreting a speaker’s illocutionary intention as a direct, indirect and highly indirect request. Therefore, children can grasp IRs both in typical development and in HFA. As for typical development, signs of understanding were visible already at age 5. This trend corroborates previous findings on pre-schoolers’ IRs comprehension (Bernicot and Legros 1987; Bucciarelli et al. 2003; Carrell 1981; Reeder 1980; Shatz 1978). As for HFAs, on the whole, children performed well in both direct and the two types of indirect requests. This result conflicts with our original prediction that if IRs understanding is compromised in HFA, then we should observe greater difficulties in the indirect and highly indirect condition as compared to the age-matched TD group. Rather, the ability to interpret an indirect request as a request seems overall preserved in HFA. We must take into consideration a possible ceiling effect in the accuracy measures, which may have limited the ability to find differences. There are two possible explanations for this. First, this can be due to the fact that we did not test very young children, in order to consider a similar age range of previous experiments and compare results. Indeed, our trend fits smoothly with previous studies, showing that HFA individuals can grasp IRs (Deliens et al. 2018, Kissine et al. 2012, 2015; MacKay and Shaw 2004). Second, other measures could be used that allow for more variation in the results also for children performing at ceiling in the task we presented. Future research should consider younger children and explore more fine-grained measures.

It must be stated here that cooperative experimental scenarios such as ours are known to enhance accuracy and compliance as compared to metalinguistic tasks. In fact, that a supportive context eases children’s interpretation of utterances is not new in the purview of developmental pragmatics. For example, young children are facilitated in metaphor comprehension and presupposition understanding within a supportive experimental scenario (Berger and Höhle 2012; Di Paola et al. 2020; Höhle et al. 2009; Pouscoulous and Tomasello 2020). Moreover, Schulze et al. (2013) found that if the paradigm is cooperative and adapted to their age even younger children show signs of understanding indirect replies—which were found to be developing only from school years (see Verbuk and Shultz 2010). In our case, the task has both act-out and metalinguistic components. It is not a classic act-out paradigm, where the accurate response to the requests would be an action. Rather, our task can be considered as an act-out task where the act is providing a response rather than an actual action. It is true that, in order to provide it, children had to perform some kind of implicit metalinguistic judgment. However, the metalinguistic component was quite limited. In fact, our task was more ecological and child-friendly than purely metalinguistic tasks such as paraphrase-choice tasks or open questions on the communicative intentions (Reeder 1980; Elrod 1987; MacKay and Shaw 2004), or story completion tasks (Bernicot and Legros 1987; Ozonoff and Miller 1996; Bucciarelli et al. 2003), which are act-out tasks but can be less ecological if they are not perceived as a game. In our case, children were engaged in the activity after a short playing session and were presented with the task as a game where the experimenter would really need their help. The experimenter could not look at the drawing she was trying to reproduce, so children arguably felt like their participation was necessary for the game to be successful. We did this trying to maintain the ecology, engagement and comfort of the act-out tasks without incurring a bias-to-action observed since the earliest studies on the development of IRs comprehension (Shatz 1978). As a consequence, the cooperative scenario might have facilitated children’s responses to the experimenter’s IRs.

As a reviewer suggests, this observation is worth considering also in light of a recent ‘interactive turn’ in social cognition research (Schilbach et al. 2013), in which the importance of practical know-how rather than propositional knowledge has been emphasized. Specifically to the case of HFA, Schilbach et al. suggest that what appears to be impaired in HFA “is not the ability to use explicit mentalistic inference, but rather, the implicit processes that contribute to participating in social interaction and that allow us to orient towards, and automatically integrate, relevant social cues in more complex situations” (2013: 411). Following this kind of reasoning, “more explicit measures of social cognition may be intact as a result of compensatory strategies” (2013: 412). With regard to this possibility, we tried to take the interactive component into account by always preferring direct communication and second-person speech in communicating with the child throughout the session. We paid particular attention to this during the task, stating that their help was crucial and we absolutely needed them or we would not be able to complete the drawing.^6^

Taking all of these observations into account, the overall high accuracy rate observed in our task can be explained. However, the three groups of participants reacted differently to the experimenter’s requests depending on whether a request was conveyed directly or not (i.e., significant Condition X Group interactions). In particular, our findings shed light on the fact that the comprehension patterns of direct and indirect requests differ in typically developing school-age children, HFA children, and typically developing pre-schoolers.

The group of older TD children showed difficulties with both indirect and highly indirect requests, while interpreting direct requests at ceiling. This suggests that, even though IRs comprehension is already developed at around 9 years of age, still an indirect or highly indirect request taxes children’s comprehension. This difficulty seems bound to the higher amount of inferential load that is involved in indirect requests and in highly indirect requests. This pattern fully confirms our specific prediction that the greater inferential complexity of a request that is not conveyed directly makes more difficult to understand a request. In turn, this is in line with earlier studies on typical development that tested the comprehension of different types of non-direct requests and found that school-age children experience extra difficulties (Bernicot et al. 2007; Bucciarelli et al. 2003).

Our HFA participants performed overall well in terms of accuracy. Yet, we observed differences between conditions. In particular, indirect requests received higher accuracy than direct requests and highly indirect requests. Let us start from the first difference: HFA children provided more correct responses to indirect requests as compared to direct requests. This finding is unexpected. In fact, one would foresee the opposite, namely a better comprehension of those types of requests whose interpretation requires simpler inferences such as, precisely, direct requests. Three possible explanations might underlie this pattern. First, the fact that HFA children’s compliance with IRs was higher than expected might be explained, as mentioned earlier, with the easing effect of the cooperative experimental scenario—and lower compliance with direct requests could simply be due to a lack of attention throughout the experimental session. Second, ASD children might tend to rely on information structure considerations, as suggested by Prévost et al. (2017). In their paper, Prévost and colleagues investigated wh-questions comprehension in French in ASD children, aged 6;3–12;9 (and a Specific Language Impairment group). Though their focus was on potential differences in comprehension of a wide variety of syntactic strategies, a major result emerged: while ASD children’s accuracy in understanding object wh-question was over 90% (even for object wh-questions with a certain degree of complexity), this was not the case for subject wh-questions. The authors interpret this as evidence for an attitude of rigid adherence to information structure considerations, since the direct object is usually the position for new information while the subject is normally used for old information. This might also have been the case for our study, where indirect requests had the requested information in the direct object position (e.g., *I don’t remember the color of the tractor*)*,* while both direct requests (e.g., *What is the color of the tractor?*) and highly indirect requests (e.g., *The color of the tractor is difficult to remember*), in which HFA children performed worse, did not.

Third, it is possible that HFAs used an alternative interpretive strategy with indirect requests that did not necessarily rely on an inferential derivation of the speaker’s illocutionary intention but, rather, used lexical clues that eased the compliance. For example, take an indirect request such as *I don’t remember the color of the tractor.* Here, the use of the word *color* alone might have clued HFAs’ response to the experimenter’s utterance, without the need to undergo a complex inferential path based on premises and conclusions about the speaker’s illocutionary intention. An explanation along these lines would be consistent with similar patterns found in the literature. For instance, Paul and Cohen (1985) found that ASD participants would heavily rely on lexical cues, which brought them to wrong responses to negative items: for example, in cases where they were told *You shouldn’t color the house blue*, they would color the house blue, since they focused on the word (i.e., *blue*) uttered by the experimenter and reacted accordingly by default, even though they did not fully process the whole utterance. In our case, a default reaction to the word *color* would lead ASD children to the right direction, i.e., to comply to our request, possibly without fully processing the whole indirect form. Moreover, Ozonoff and Miller (1996) found that ASD individuals interpreted more often a request as indirect than direct even in a context that supported the interpretation of the utterance as a question (e.g., *Can you water the lawn?* uttered in a conversation about lack of water resources in town). The authors explain ASD individuals’ preference for the indirect interpretation as mirroring either an impairment in using context and meaning appropriately or an overgeneralization process by which ASD individuals associated the *Can you…*? form to a request by default, independently of context. Beyond this, it has been suggested that ASD individuals might not hinge on complex interpretive strategies for IRs understanding as compared to neurotypical controls (Kissine et al. 2012)—see also Paul and Cohen (1985). The idea of HFA individuals’ use of an interpretive strategy that is based on linguistic clues could also explain the second pattern that emerged in this group, namely more correct responses to indirect than highly indirect requests as well. In fact, while hinging on lexical clues might have been enough to comply with an indirect request, the same might have not hold for a highly indirect request. Highly indirect requests such as *The color of the grass is hard to remember* are intuitively more obscure than indirect requests (e.g., *I don’t remember the color of the grass*), for several reasons that include the wording of the utterance and the greater inferential work involved. It is possible that lexical clues alone were not sufficient enough to support HFA individuals’ compliance with this type of request.

An even different comprehension pattern emerged in the group of younger TD children. On the one hand, they showed an understanding of the requests (i.e., high accuracy rates). On the other hand, no differences among conditions emerged, thus suggesting that the younger TD children responded similarly to a request be it direct, indirect or highly indirect. In other words, it seems that the neurotypical preschoolers were not sensitive to the manipulation and that, independently of the complexity of the inferential derivation process, they complied equally to all types of requests. What can explain this pattern? We interpret this finding along the lines of a simplified comprehension strategy that, similarly to the case of the HFA group, used contextual and linguistic clues to successfully comply with the requests. In our design, at least two factors might have supported younger children’s comprehension. First, as mentioned earlier, the general experimental setting was a cooperative game in which the children were instructed to help the experimenter recreating a drawing. This might have enhanced their performance independently of the type of request—i.e., children might have inferred that, whatever the specific phrasing of the requests, they would inform the experimenter about the drawing. Second, similarly to HFA children, younger TD children might have relied on some linguistic cues—for instance the word *color*—to comply with the request, without paying much attention to the specific formulation of the request. This explanation fits nicely with previous findings, showing that preschoolers tend not to be influenced by the type of request, but rather their interpretation hinges on context regardless of the linguistic form (Bernicot and Legros 1987). Future research should explore this issue further.

Overall, the group differences discussed above suggest that children understand IRs, in HFA as well as in typical development, both during school and preschool years. Importantly, they might rely on different interpretive strategies. During school years, neurotypical children seem to rely on a genuinely inferential strategy and to deal with the corresponding complexity (i.e., they show more difficulties with (highly) indirect than direct requests). Conversely, when the cognitive functioning is more limited, either because a developmental disorder such as HFA or because of the specific developmental phase (i.e., preschool years), children might rely on simpler strategies based on linguistic clues rather than a full inferential derivation of IRs.

Our findings only partially replicate Kissine et al. (2015). Similarly to Kissine et al. (2015), we suggest that HFA individuals’ ability to understand IRs is preserved. However, contrary to Kissine et al. (2015), we cannot conclude that HFA individuals were even more competent than TD children in IRs understanding. As we have shown, when compared to an age-matched group of TD peers, HFA children do not perform better than TD children. This casts some light on two main points. First, HFA children’s better performance than TD children in Kissine et al.’s study likely has to do with a developmental bias. Second, more importantly, in comparing HFA children to age-matched TD controls, we have shown that different interpretive strategies actually enhance IRs understanding in these samples of participants. In addition, Kissine et al. hypothesized that a lack in ToM abilities might have explained their young TD children’s lack of IRs comprehension. Our results do not corroborate such a hypothesis. We collected measures for the participants’ mind-reading skills and results suggested that the younger TD children exhibited mind-reading skills that were similar to HFAs. Additionally, ToM did not significantly predict IRs understanding in either of the two groups (more on this later). Again, the differences emerged in Kissine et al. likely reflect more general developmental effects.

As expected, children’s mind-reading abilities were not the same in all groups: the group of older TD children exhibited overall better ToM skills than both HFA children and the younger TD group, as shown by significant group differences in first-order ToM and in ToM composite scores. The difference between the HFA group and both older and younger TD children in the second-order ToM task only approached significance, probably due to small sample sizes (children who did not pass the first-order ToM task were not presented with the second-order one).

Most importantly, for the purpose of our study, the analysis of predictors revealed that the children in the older TD group who showed better performance in ToM also showed higher accuracy in the comprehension of highly indirect requests. Contrary to HFAs and the TD preschoolers, ToM seems to enhance the older TD children’s understanding of highly indirect requests. Moreover, this result further corroborates the hypothesis that not only different interpretive strategies might take place in the three groups of children, but also only the older TD children genuinely rely on an inferentially driven strategy. To the best of our knowledge, this is first evidence that ToM is directly involved in IRs comprehension in (a)typical development. Any hypothesis on ToM involvement in IRs comprehension in autism is difficult to articulate because we also do not have a clear picture of the role of ToM in understanding IRs in the neurotypical population, yet. In this respect, it is worth mentioning that consistent (though not directly comparable) findings about the involvement of ToM come from some studies that investigated the role of the broader autism phenotype^7^ in adults’ processing of pragmatic information—which is overall related to an involvement of ToM abilities in pragmatics. For instance, Nieuwland et al. (2010) found participants’ variations in N400 responses based on their autistic traits: individuals that presented higher autistic traits were less sensitive to pragmatic violations and exhibited no pragmatic N400 effect. Similarly, Mazzaggio and Surian (2018) suggested that a weaker tendency to draw pragmatic implicatures is linked to autistic traits.

The involvement of ToM in IRs comprehension that emerges in our results is therefore worth investigating further, and at least two relevant questions can be put forward. Why did ToM significantly predict only the comprehension of highly indirect requests? Why only for older TD children? If we accept that highly indirect requests represent the most compelling condition in terms of processing, then it seems reasonable to observe that mind-reading skills are prominently involved in the comprehension of highly indirect requests in a behavioral task. In fact, for successful interpretation, they are expected to require more complex inferential steps in order to recognize the speaker’s illocutionary intention, as compared to direct and indirect requests. Interestingly, this pattern emerged only with the older TD children. If, on the one hand, this is surprising, on the other hand, this fits perfectly with the idea that the different groups relied on different interpretive strategies. The reason is that, in the analysis of predictors, a main effect of ToM, i.e., for all groups and conditions, approached significance. However, this emerged more prominently with the group of older TD children only. It is interesting to observe that older TD group were also the only one who neatly discriminated between direct and non-direct requests (of the two kinds): younger TD children performed similarly in all conditions, HFA children performed similarly in direct and highly indirect requests, while older TD children performed worse with non-direct requests (i.e., indirect and highly indirect) than with direct. This allows us for a speculation. In our design, the type of request influenced older TD children’s accuracy rates. Hence, only older TD children appeared to be sensitive to the different amount of inferential work needed to reconstruct the speaker’s illocutionary intention in direct vs. non-direct requests. Furthermore, older TD children also exhibited better ToM abilities than younger TD children and HFA children. Therefore, it might be the case that older TD children—and not the younger TD group, nor the HFA group—exploited their ToM skills at their best to deal with the indirectness of the requests. In other words, older TD children might have genuinely used a mind-reading-based interpretative strategy to derive the contextually adjusted speaker’s illocutionary force. Differently, younger TD children and HFA children might rely on different strategies based on linguistic clues; this is not new in the literature of ASD and pragmatic skills. Indeed, in a recent work, Hochstein et al. (2017) have shown that adolescents and children with ASD can compute pragmatic inferences, but they do so also in contexts in which they are not expected to. Particularly, ASD participants in their study accomplished with the task without reasoning on the epistemic states of their interlocutors.

Several recent studies also support the idea that pragmatic processes in autism may rely on different strategies than in neurotypical individuals. For example, Ostashchenko et al. (2020) found that ASD children could perform well in a selective trust task, where one speaker would consistently misname familiar objects. Both TD and ASD children avoided information provided by the previously inaccurate speaker. Neurotypical children would probably create epistemic models of speakers to perform the task, which in turn would require certain levels of social understanding that ASD children would not be expected to show. However, the authors believe that ASD children might have relied on a simple associative process, rather than a proper epistemic model. Something similar can be observed in a recent paper by van Tiel et al. (2020), where ASD individuals were tested in a task on deception, which is normally considered as an indicator of perspective taking. However, ASD participants proved equally likely than TD participants to detect deception. The authors explained this finding as an indicator of possibility that ASD individuals relied on a different strategy not involving perspective-taking, i.e., deriving regularities from behaviors observed throughout the experiment, to compensate for their difficulty with perspective-taking (see also Livingston et al. 2019). This hypothesis is interesting and would require further investigations.

## Conclusions

The main take-home message of this study is that: first, IRs understanding seems preserved in HFA; second, unlike age-matched TD children, HFA children seem to employ an interpretive strategy that is not mainly based on deriving the speaker’s intended illocution; third, mind-reading skills support the comprehension of highly unconventional IRs in typically developing children during school years (and perhaps, for IRs in general, they rely on a mind-reading interpretative strategy). Assuming that TD and HFA children actually interpret IRs differently, the next step is to disentangle which pragmatic strategies are at play with IRs and whether these may vary depending on language users’ individual features. Recently, Andrés-Roqueta and Katsos (2017, 2020) proposed the distinction between Linguistic- and Social-Pragmatics. In this view, Linguistic-Pragmatics includes those pragmatic tasks whose comprehension relies on the hearer’s egocentric point of view: linguistic abilities (e.g., lexical and morphosyntactic competence) and basic knowledge of pragmatic norms would suffice to succeed a linguistic-pragmatic task (e.g., scalar implicatures and sensitivity to informativeness). Conversely, Social-Pragmatics includes those pragmatic tasks whose comprehension depends mainly on perspective-shifting skills: linguistic abilities and knowledge of the basic pragmatic norms underdetermines the comprehension of a pragmatic task for which inferring the speakers mental state is essential (e.g., irony, creative metaphors, etc.).

Researchers in this field generally agree that pragmatic interpretation can involve different strategies. Most of the debate, up to now, focused on the idea that this variation is bound to the specific pragmatic phenomenon as well as to the experimental task adopted. Recently, an increasing wealth of studies addressed the timely issue of individual variation in pragmatics. Gibbs and Colston (2012), for instance, suggest that beyond experimentally related factors, several individual features such as age, clinical and social status, affect the understanding of a pragmatic phenomenon like figurative language. In addition, not only it has been already demonstrated that the development of metaphor comprehension depends on individual variation (Carriedo et al. 2016; Di Paola et al. 2020; Pouscoulous 2014), but some researchers made also the point that interpersonal factors likely play a role in the development of several pragmatic abilities (Matthews et al. 2018). Overall, then, it seems plausible that different interpretative strategies might be followed for the same pragmatic phenomenon, depending on individual factors. Our study opens to the possibility that HFA children rely more on strategies pertaining to the Linguistic-Pragmatics realm to understand IRs, while TD children rely more on strategies associated with Social-Pragmatics. Future studies should verify this hypothesis.

## Appendix

See Appendix Tables

**Table 5:** Studies on IRs comprehension in TD children

Author (year)	*N*, group, age range	Task	Comparison	Main findings	Interpretation
Shatz (1978)	18 TD, 1;7–2;10	Requests for action and for information of 8 sentence types (e.g., *Can you…?; Why don’t you…?; Would you…?*) on different toys	For each sentence type, verbal versus action responses (on children’s total meaningful responses and on total responses)	For all sentence types, actions were 91% of the meaningful responses and 62% of all responses	Two-year-olds grasp interrogative IRs Children are strongly biased to respond to language with action, regardless of its form
Reeder (1980)	7 TD, 2;6 7 TD, 3;0	Paraphrase-choice task; *Would you like to x?,* in 2 contexts, that supported an offer (*I’ll let you x*), or an IR (*I want you to x*) interpretation	Effect of condition: indirect offers versus interrogative IRs. Effect of age group Interaction condition X age group	Significant condition X age group interaction: 3-year-olds performed better than 2; 6-year-olds in the IR condition (but not in the indirect offers condition)	Children between 2; 6 and 3 years grasp interrogative IRs. However, interrogative IRs are more difficult than indirect offers, possibly because younger children are more exposed to offers than to IRs
Carrell (1981)	21 TD, 4;0 30 TD, 5;0 25 TD, 6;0 24 TD, 7;0	Children knew they would be asked to color a circle either in red or in blue Linguistic material: 10 pairs of IRs of different forms (declaratives; interrogatives; N.2 imperatives) with positive vs. negative surface polarity (e.g., *You should/shouldn’t color…; Should/Shouldn’t you…?)*	For each pair, differences in proportions between age groups Differences in proportions between interrogatives and declaratives For each pair, differences in proportions between negative and positive surface polarity items	Accuracy by age group: 4 64.5% at 4; 73.5% at 5; 78% at 6, 92% at 7 Interrogatives were more difficult than declaratives Negative conveyed IRs were more difficult than positive counterparts	Children are able to comprehend a wide variety of IRs, but they are influenced by surface polarity There is a general developmental pattern, but IRs are not fully acquired before age 7
Bernicot and Legros (1987)	24 TD, 3;0–4;1 24 TD, 5; 1–5;11	DIRs, IRs, and non-directives were presented in stories with a weakly or strongly supportive context. A character would not comply with the request. Children had to choose whether the speaker was angry, unhappy, or okay	Effect of age group Effect of condition: non-directive vs DIR versus IR Effect of context: strong versus weak Interactions age X condition X context	Older children performed better than younger children. They interpreted directively DIRs more than IRs, and IRs more than non-directives. They correctly interpreted IRs more often when the context was strong	Children at 3–4 years of age seem not to perceive condition nor context manipulation Children at 5–6 years of age do, but IRs comprehension is not fully acquired yet
Elrod (1987)	39 TD, 3;3–4;7 39 TD, 4;8–6;5	Children presented with stories with a DIR or a HIR (e.g., *Those cookies are for our guests*) and were asked: (i) questions about intentions; (ii) to choose a picture to complete the stories	Effect of age group Effect of condition: DIR versus HIR Interaction age X condition	HIRs were more difficult to understand for both groups. Older children performed better than younger children in (i) for IRs and in (ii) regardless of condition	The two age groups mostly differ in IRs comprehension, while their comprehension of DIRs is similar Children younger than 4; 7 do not understand IRs as well as DIRs
Bucciarelli et al. (2003)	40 TD, 2;6–3;0 40 TD, 3;6–4; 0 40 TD, 4; 6–5;6 40 TD, 6;0–7;0	Children asked to complete stories including IRs (e.g., *Sorry, could you close the window?*), HIRs (e.g., *Excuse me, I am studying*), and DIRs—among several pragmatic phenomena These were presented in both a linguistic and a gestural protocol	Effect of condition, 5 levels: Directs, Simple Indirects (including IRs), Simple Deceits, Simple Ironies, Complex Indirects (including HIRs) Effect of condition by age group Effect of protocol: linguistic versus gestural	Effect of condition: Simple Indirects were easier than Complex Indirects Effect of condition by age group: a developmental pattern emerged for Complex Indirects: at 2;6–3 (38%); at 3;6–4 (42%) and 4;6–5;6 (43%); at 6–7 (68%) No difference between protocols	Complex Indirects such as HIRs are still developing at 6–7 years of age. This may be due to both the complexity of the mental representations and the inferential load involved

TD: typically developing; IRs: Indirect requests; HIRs: Highly indirect requests; DIRs: Direct requests

5,

**Table 6:** Studies on IRs Comprehension in ASD

Author (year)	*N*, group, age range or mean	Task	Comparison	Main findings	Interpretation
Paul and Cohen (1985)	8 ASD adults mean age: 22.3 8 non-ASD cognitively disabled adults mean age: 27.9	Adapted from Carrell (1981; see Table 1), with the addition of 2 different conversational scenarios: structured or unstructured	Effect of: group; Scenario: structured vs. unstructured Sentence type: interrogative versus declarative Surface polarity: positive versus negative conveyed IRs Interactions: group X scenario X sentence type; group X scenario X surface polarity	Effect of group: ASD adults performed worse than controls in both scenarios Effect of scenario X group: ASD adults performed worse in the unstructured than in the structured scenario. Interaction of scenario X group X sentence type: in the unstructured scenario, negative IRs were more difficult than positives for ASD adults	Both groups performed similarly to TD children in Carrel (1981). ASD participants show a pragmatic impairment that makes it more difficult for them to comply with IRs in an unstructured scenario
Ozonoff and Miller (1996)	17 HFA 16;1–57;8 17 NT adults 16;5–45;2	Participants asked to choose the response to *Can you…?* forms in 2 conditions: the context suggested interpretation as direct questions or as IRs. (2 other tasks also tested humor and inference.)	Effect of group Effect of condition: direct question versus IR Interaction group X condition	HFA participants chose significantly more IR than direct responses, regardless of condition Main effect of group: HFA participants provided overall less correct responses than NTs. Main effect of condition: IR was overall easier than direct question	Results suggest a specific underlying impairment in using context for IRs interpretation in HFA, rather than a tendency to be overly literal
Deliens et al. (2018)	24 ASD, 15–52 24 NT, 15–53	Participants heard *Can you…?* and *Is it possible to…?* sentences (and control imperatives and interrogatives) about moving shapes in a grid. If sentences were interpreted as IRs, participants should move the shapes; in case of non-directive interpretation, they should click on *yes*/*no* buttons. Response times and eye fixations on the buttons were also collected	Effect of group Effect of condition: *Can you…?* versus *Is it possible to…?* Interaction group X condition Effect of condition in the Response Time (RT) analysis: control imperatives and interrogatives were also taken into account	Effect of group X condition: NT participants interpreted *Is it possible to…?* forms directively less than they did for *Can you…?* forms Effect of group in the RT analysis: NT were faster than ASD participants. Effect of group X condition: ASD participants took longer than the NT group in providing a literal response to *Can you…?* forms	ASD adults have no difficulties with interrogative IRs comprehension (while they have difficulties with irony—tested by the authors in a separate task). The author report that it is not clear why ASD adults interpreted *Is it possible to…?* forms directively more than the NT group
MacKay and Shaw (2004)	19 HFA children, 8;0–11; 7 21 TD children, 9;0–10;11	Children heard stories ending with HIRs and were asked one question about the “meaning” of the utterance and one about its “intent”. Hyperbole, irony, metonymy, rhetorical questions, understatement were also tested)	Effect of group	HFA and TD children correctly understood the meaning of IRs. Effect of group: HFA participants exhibited more difficulties at explaining the intent	HFA participants could understand the meaning of IRs, but they had poor understanding of a speaker’s intention and motives in using an IR
Kissine et al. (2012)	11 ASD children, 4;3–12;5	Children asked requests through 4 sentence types (imperative, declarative, interrogative, sub-sentential)	Effect of sentence type: imperative versus declarative versus interrogative versus sub-sentential	ASD children complied well with all types of requests (no significant differences)	HFA children seem to comply with IRs. Their interpretive strategies might be fairly simple, given their very low IQ
Kissine et al. (2015)	15 ASD children, 7–12 20 TD children, 2;7–3;6	Children asked to “dress” a toy with a hat using the HIR: *Oh, he has no hat!* (phase 1). The same sentence was uttered as a comment by another speaker (phase 2) and by the first speaker (phase 3)	For each phase, effect of group	Phase 1: ASD children performed at ceiling; TD children performed significantly worse than ASD children Phases 2 and 3: No significant group differences	IRs comprehension seems to be preserved in ASD. TD children had difficulties, instead; these results might be due to development in ToM

ASD: Autism spectrum disorders; HFA: High-functioning autism; NT: Neurotypical; TD: Typically developing; IRs: Indirect requests; HIRs: Highly indirect requests

6,

**Table 7:** Studies on ToM and IRs Comprehension

Author (year)	*N*, group, age range or mean	Task	Comparison	Main findings	Interpretation
van Ackeren et al. (2012)	16 NT, mean age: 21.39	fMRI study; HIRs (e.g., *It is very hot in here*) presented with images biasing toward a literal (e.g., a desert) or IR interpretation (e.g., a window). False-belief/localizer task to identify the ToM and cortical motor network	Effect of condition: IR versus literal versus utterance control versus picture utterance control condition ROI analysis and whole-brain analysis were performed	Effect of condition: HIRs triggered a stronger BOLD response than controls in regions identified as part of the ToM network (medial pre-frontal cortex and temporo-parietal junction)	Understanding IRs requires a similar inference on the mental state of the speaker as ToM tasks
Trott and Bergen (2018)	Exp 1: 42 NT, mean age: 37 Exp 2: 83 NT, mean age: 33 Exp 3: 84 NT mean age: 34.3	Exp 1–2: Paraphrase-choice task; Exp 3: request judgment task (Yes/No). In the stories material, the speaker’s awareness was manipulated: the speaker could either be aware or unaware of an obstacle to the fulfillment of their HIR Short story task for ToM (Exp 2–3)	Effect of condition: Speaker aware versus speaker unaware Effect of predictor: ToM task Interaction condition X ToM	Main effect of ToM and interaction with speaker awareness: higher ToM abilities enhanced participants’ ability to recognize IRs when the speaker was unaware of the obstacle	ToM abilities seem to predict whether the speaker’s inferable knowledge states are taken into account during the comprehension of IRs
Cuerva et al. (2001)	12 AD with ToM 70.6 22 AD without ToM 72.5 10 NT mean age: 60.6	14 AD participants and the control group were presented with 10 vignettes: 5 eliciting IRs production, 5 assessing comprehension of conversational implications. The comprehension part was a paraphrase-choice test. All 34 participants were also presented with a second-order ToM (false belief) and Short Stories tests	Effect of group: (7) AD patients with ToM versus (7) AD patients without ToM versus (10) controls	Main effect of group: both groups of AD performed worse than controls in both production and comprehension. In the comprehension task, patients without ToM performed significantly worse than patients with ToM, as well	AD patients with mild dementia present deficits in both ToM and pragmatic abilities and there seems to be a relationship between the two, as AD patients without ToM showed significantly more deficits than AD patients with preserved ToM
Champagne-Lavau and Joanette (2009)	15 adults with RHD, mean age: 60.9 15 NT adults, mean age: 60.7	Participants were presented with utterances within a context implying a literal or a HIR interpretation. If they failed when asked to explain, they would get a multiple-choice cue. They also performed a ToM (false belief) task. (Metaphor and executive functioning were also assessed)	Effect of group Effect of condition: literal vs. HIR Interaction group X condition Hierarchical cluster analysis on IRs task and metaphor task performances of the patients	Effect of group: patients performed overall worse than controls in both the IRs task and the ToM test. Effect of condition: participants of both groups performed worse in the indirect condition. In the clusters of patients identified as impaired in indirect conditions, this co-occurred with first-order ToM (and inhibition) difficulties	Patients with right-hemisphere lesions have impairments in IRs comprehension and ToM abilities; the ability to understand IRs is closely associated with ToM abilities
Muller et al. (2010)	15 adults with TBI, mean age: 37.2 15 NT adults, mean age: 37	Participants were presented with stories ending with DIRs or HIRs and had to explain what the speaker meant. A ToM battery was also administered (false beliefs, Faux pas, character intention task, Reading the Mind in the Eyes). Empathy and executive functioning were also assessed	Effect of group Effect of condition: DIR versus IR Correlation between ToM tasks and IRs task	Effect of group: patients performed significantly worse than controls in the IRs task, as well as on all ToM tasks, except for the first-order task. A high correlation was found between the IRs task and ToM tasks (all but the Reading the Mind)	Participants with traumatic brain injury might have problems in using context to understand both IRs and DIRs, as well as mentalizing questions

NT: Neurotypical; AD: Alzheimer’s disease; RHD: Right hemisphere damage; TBI: Traumatic brain injury; IRs: Indirect requests; HIRs: Highly indirect requests; DIRs: Direct requests

7.

## Footnotes

1 Relevance Theory initially rejected the idea of an autonomous module for pragmatic processing. Relevance theorists argued that pragmatic interpretation is a process based on the central system of thought. Sperber and Wilson (2002) revised their position on modularism arguing for a specific module devoted to pragmatic interpretation, which is the product of the evolution of the human capacity for ‘mind reading.’

2 More recently, other accounts have been proposed which bind the pragmatic impairment in ASD to a more general linguistic and cognitive impairment—i.e., not only an impairment in ToM. For instance, the Weak Central Coherence hypothesis attributes these difficulties to ASD individuals’ tendency to focus on small—rather than global—pieces of information as a result of the failure of a central system that integrates information and context (Frith 1989; Happé and Frith 2006). The executive dysfunction account, instead, links these difficulties to a lack of cognitive flexibility and/or inhibition (Hill 2004; Kissine 2012).

3 Interestingly, a recent study by Schaeken et al. (2018) suggested these results might be due to the task used. In fact, using a classic statement evaluation task where ASD children and controls could express their judgement on underinformative utterances on a ternary option (I agree vs. I agree a bit vs. I disagree), ASDs tended to either fully agree or fully disagree with underinformative statements, while TD children preferred the middle answer option. Crucially, autistic participants’ performance in scalar implicatures also seems to differ from neurotypical participants’ when implicature derivation requires representing the speaker's beliefs (Hochstein et al. 2017).

4 Data were previously analyzed with Generalized Linear Mixed Models (GLMMs). However, even proceeding from a maximal to a more parsimonious random-effect structure, the models did not converge, likely because there was not much variance in the random effects. After this, logistic regression was used, but the regression models were highly affected by multicollinearity. To resolve the multicollinearity issue, participants’ accuracy in the IRs task was transformed in composite scores, which allowed for LMMs statistics.

5 This was done because, as mentioned before, previous GLMMs did not converge and the subsequent logistic regression models were highly multicollinear.

6 We acknowledge that more interactive tasks could also be thought of for mind-reading abilities and that further research would be needed in this respect, from an interactionist point of view. For a critique of the interactive turn in social cognition research, see Overgaard and Michael (2015).

7 This is a phenomenon that refers to the presence and distribution of autistic traits in the general population.

## Declarations

### Availability of data and material

Data and material are available in the Supplementary Material hosted on the Open Science Framework web platform (link to the project: https://osf.io/p2dn5).

### Code availability

Code is available in the Supplementary Material hosted on the Open Science Framework web platform (link to the project: https://osf.io/p2dn5).
